# A novel pathogenic variant of the *FH* gene in a family with hereditary leiomyomatosis and renal cell carcinoma

**DOI:** 10.1038/s41439-021-00180-8

**Published:** 2022-01-17

**Authors:** Yasuto Yagi, Naoko Abeto, Junichi Shiraishi, Chieko Miyata, Satomi Inoue, Haruka Murakami, Moeko Nakashima, Kokichi Sugano, Mineko Ushiama, Teruhiko Yoshida, Kazuki Yamazawa

**Affiliations:** 1 Department of Urology, National Hospital Organization Tokyo Medical Center, Tokyo, Japan; 2 Department of Pathology, National Hospital Organization Tokyo Medical Center, Tokyo, Japan; 3 Department of Pathology, National Cancer Center Hospital, Tokyo, Japan; 4 Department of Palliative Care Medicine, National Hospital Organization Tokyo Medical Center, Tokyo, Japan; 5 Medical Genetics Center, National Hospital Organization Tokyo Medical Center, Tokyo, Japan; 6 Department of Genetic Medicine, Sasaki Foundation, Kyoundo Hospital, Tokyo, Japan; 7 Department of Genetic Medicine and Services, National Cancer Center Hospital, Tokyo, Japan

**Keywords:** Renal cell carcinoma, Cancer genetics, Genetics research

## Abstract

Hereditary leiomyomatosis and renal cell carcinoma caused by loss-of-function germline variants of the *FH* gene can develop into aggressive renal cell carcinoma (RCC). We report the case of a 27-year-old man who died of RCC. Genetic testing revealed a novel pathogenic variant of *FH*, NM_000143.3:c.1013_1014del (p.Ile338Serfs*3), that was also identified in healthy siblings. Identification of genetic causes in the proband helped us to provide relatives with precise genetic counseling and appropriate surveillance programs.

Hereditary leiomyomatosis and renal cell carcinoma (HLRCC) is a newly emerging hereditary tumor syndrome with an autosomal dominant inheritance pattern characterized by cutaneous leiomyoma, uterine leiomyoma, and renal cell carcinoma (RCC)^1^. More than 300 families with HLRCC have been reported to date, although the condition may be underdiagnosed^2,3^. Loss-of-function germline variants of the *FH* gene, which is located on chromosome 1q43 and encodes fumarate hydratase, cause HLRCC^4^. The incidence of RCC in *FH* pathogenic variant carriers is estimated to be approximately 15–20% with a median age of onset of 35–44 years^3,5,6^; however, a substantial number of *FH* pathogenic variant carriers can develop RCC at a very young age. Actually, a case of a seven-year-old boy with HLRCC-associated RCC was recently reported^7^. Histologically, these renal tumors are characterized by a unique type 2 papillary architecture but can also have a variety of patterns, such as cystic, tubular, solid, or mixed patterns^8^. HLRCC-associated RCC is typically aggressive and metastatic, and most patients with this type of RCC are at an advanced stage at the time of diagnosis. Thus, surveillance and early detection are of great importance^9^. Herein, we report a familial case of HLRCC caused by a novel pathogenic variant of *FH*.

A 26-year-old Japanese man was admitted to our hospital with right inguinodynia and motor dysfunction of the right lower limb. His medical history was unremarkable. Notably, his mother died of cancer of unknown primary origin at the age of 41 years, nine years before his admission. She had pelvic tumors with multiple metastatic lesions in the lungs, skin, left kidney, and vertebra. Malignant transformation of uterine leiomyomas was suspected, but an accurate assessment of the primary origin was not provided. The patient’s maternal grandfather died of pulmonary adenocarcinoma at the age of 74 years, and none of the other family members had HLRCC-associated episodes (Fig. 1a).

**Fig. 1:**
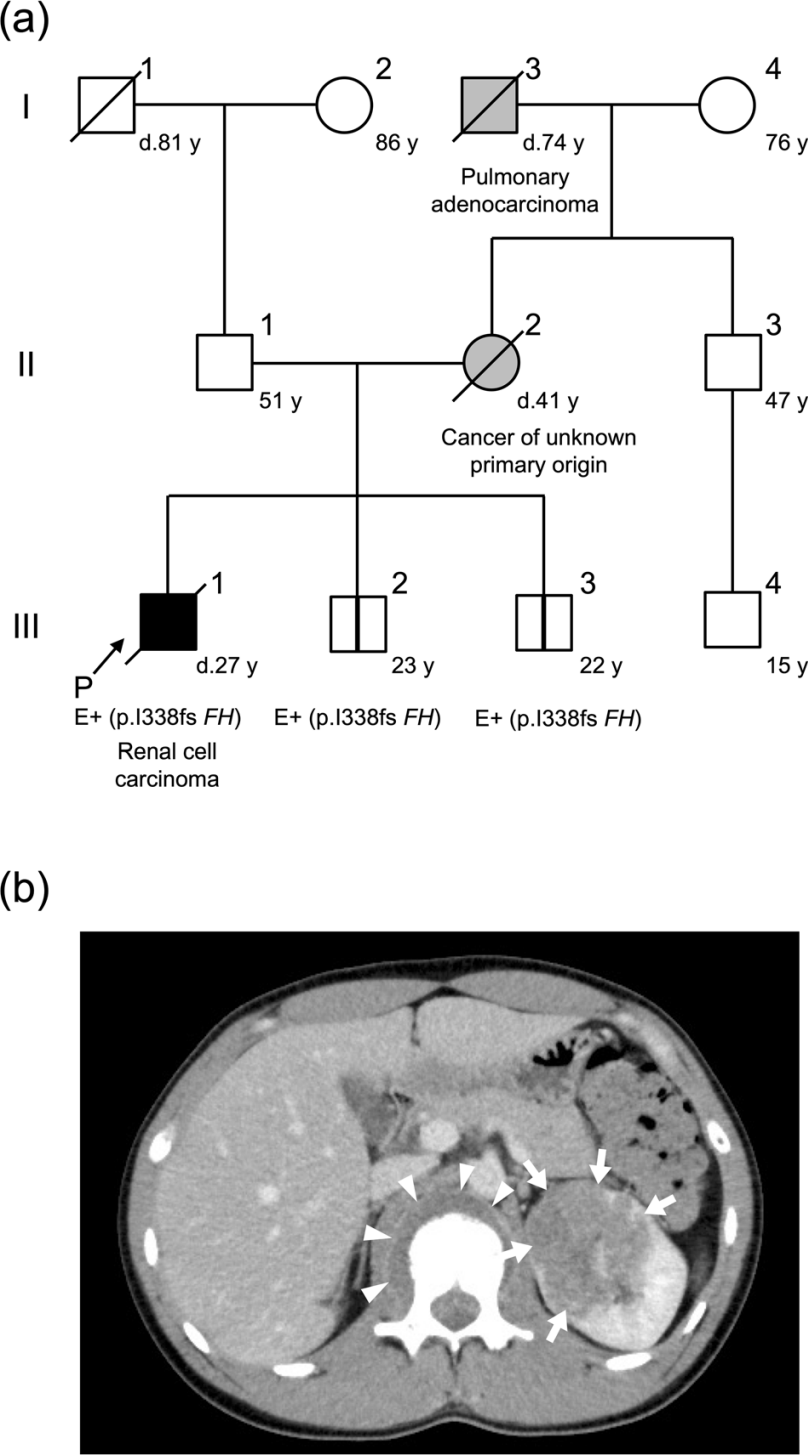
Pedigree and radiologic findings of the patient. **a** The patient (III-1) died of renal cell carcinoma at the age of 27 years. His mother (II-2) died of cancer of unknown primary origin at age 41 years. The pathogenic variant of *FH* was identified in the patient as well as his two younger brothers (III-2 and III-3). P, proband; Roman numerals represent generation numbers; Arabic numerals indicate individual numbers; females are represented by circles and males by squares; E+ denotes the *FH* variant carrier; vertical bar denotes asymptomatic variant carrier. **b** Abdominal computed tomography revealed a solid mass in the left kidney (arrows) and an adjacent paravertebral tumor (arrowheads) of the patient.

On admission, the patient’s laboratory tests were normal, except for mildly elevated levels of C-reactive protein (0.69 mg/dL) and lactate dehydrogenase (617 U/L). He did not develop any cutaneous manifestations. Enhanced computed tomography scanning revealed a solid mass measuring approximately 60 mm in the left kidney and an adjacent paravertebral tumor (Fig. 1b), coupled with multiple pulmonary nodules and osteolytic lesions. Although pathological examination was not available at this point, RCC was suspected with TNM stage cT4NxM1. The patient was considered inoperable; thus, combination therapy with the immune checkpoint inhibitors nivolumab and ipilimumab was initiated. After the second administration, ipilimumab was intolerable due to the exacerbation of immune-related adverse effects. Subsequent treatment with nivolumab alone was continued but found to be ineffective. Thus, pazopanib and cabozantinib were administered with palliative radiation therapy.

Given the early onset of cancer with an aggressive phenotype in the patient as well as his mother, we proceeded to conduct genetic investigations as described in the Supplementary Methods after providing genetic counseling and obtaining written consent. Germline multigene panel testing identified a novel heterozygous frameshift variant NM_000143.3:c.1013_1014del (p.Ile338Serfs*3) in the *FH* gene in the patient, thereby confirming the diagnosis of HLRCC-associated RCC. A multiplex ligation-dependent probe amplification (MLPA) assay revealed no copy number changes in *FH* (data not shown).

Thereafter, the tumor continued to progress, and the patient eventually died 13 months after the initiation of treatment. An autopsy confirmed an invasive substantial tumor measuring 45 × 25 × 15 mm at the upper part of the left kidney (Fig. 2a). Microscopically, the tumor exhibited a predominantly papillary growth pattern (Fig. 2b). At a higher magnification, clear and eosinophilic tumor cells possessed large nuclei with eosinophilic and enlarged nucleoli surrounded by a halo (Fig. 2c), which is the histological hallmark of HLRCC-associated RCC^10^. Immunohistochemistry for FH revealed minimal staining (Fig. 2d), demonstrating FH-deficient RCC. Additional Sanger sequencing analysis of the *FH* variant using blood- and tumor-derived DNA showed that the mutant allele was predominant in the tumor (Fig. 2e), suggesting loss of heterozygosity (LOH).

**Fig. 2:**
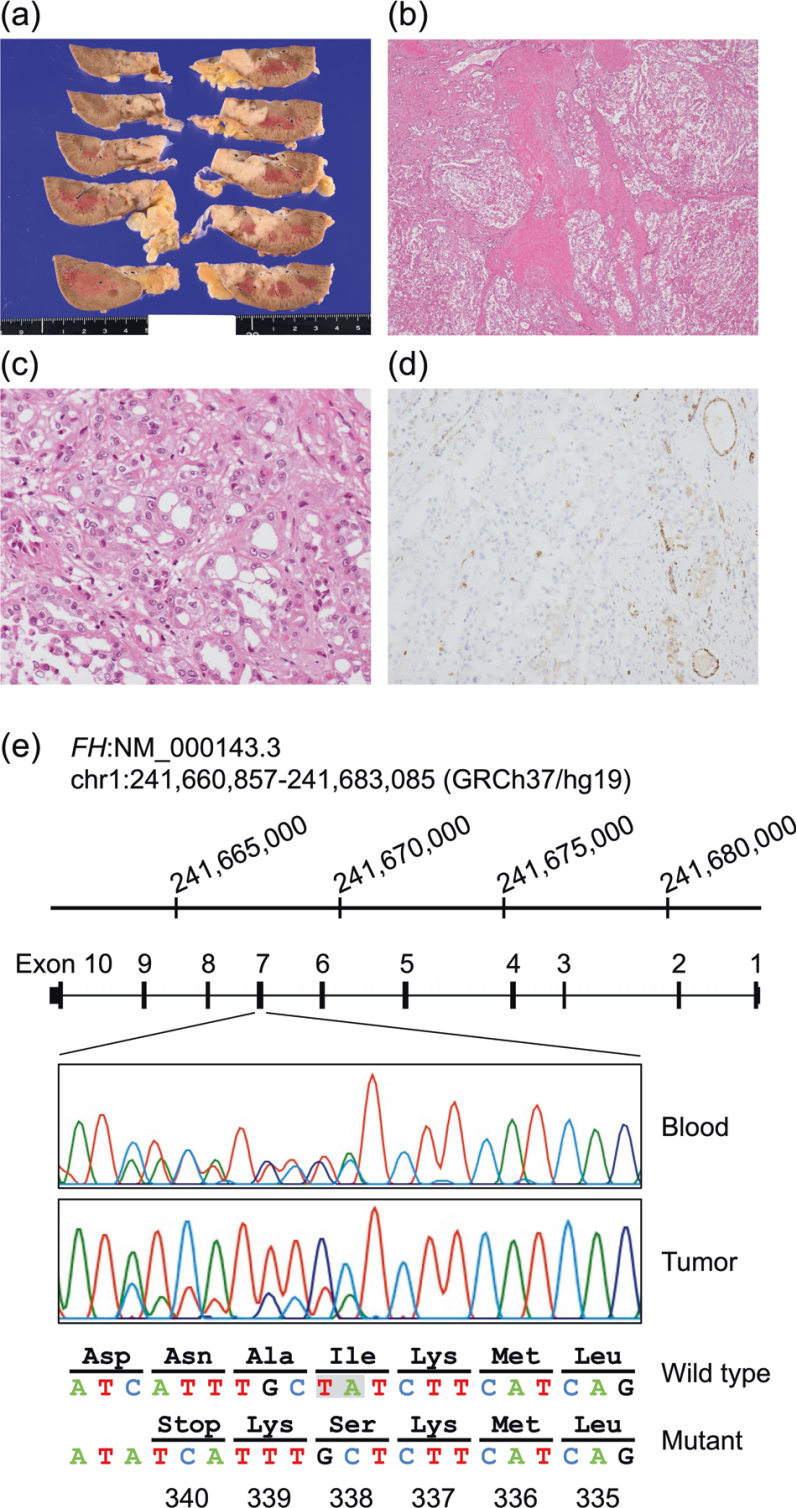
Histopathological and sequencing findings of the patient. **a** Surface section of the left kidney. A yellowish-white solid tumor was observed. **b** Hematoxylin and eosin staining of the tumor demonstrated a predominantly papillary architectural pattern. **c** Higher magnification of the tumor showed that clear and eosinophilic tumor cells possessed large nuclei with eosinophilic and enlarged nucleoli surrounded by a halo. **d** Immunohistochemistry for FH was almost negative. **e** Schematic presentation of the *FH* gene (NM_000143.3) and pathogenic variant identified in this study. Sanger sequencing data of the *FH* gene confirmed a frameshift variant c.1013_1014del (p.Ile338Serfs*3) in the blood and tumor samples of the patient (highlighted by a gray box). Of note, the mutant allele was predominant in the tumor, indicating loss of heterozygosity.

Two younger siblings of the patient aged 22 and 23 years, who had no remarkable medical history, decided to undergo genetic testing after genetic counseling sessions. The results demonstrated that both had the same *FH* variants. At that time, no renal tumors or cutaneous manifestations were noted in the siblings. Hence, annual surveillance with magnetic resonance imaging for HLRCC-associated RCC and regular skin check-ups were scheduled.

The clinical course of the mother was reminiscent of HLRCC, although genetic testing had not been conducted. Given that two siblings shared the *FH* variant with the proband, the mother was assumed to harbor the same variant and be affected by HLRCC-associated malignancy. However, it is unclear whether the variant emerged de novo in the mother or was inherited from the maternal grandparents. In this regard, 20 Japanese families of HLRCC have been reported to date, and different *FH* variants have been reported in these families (Supplementary Table S1). The variants are presumed to be independent of each other; thus, a founder effect in the Japanese population is less likely.

The identified germline *FH* variant c.1013_1014del (p.Ile338Serfs*3) has hitherto not been reported. The variant is predicted to cause a frameshift in exon 7 of the *FH* gene, which can result in truncation of downstream amino acids. Of note, analysis of the somatic mutations of the tumor did not reveal any other pathogenic variants in the *FH* gene but revealed an LOH pattern. Specifically, the wild-type allele was lost, and the germline variant was retained. The occurrence of such an LOH event in the *FH* gene, which is frequently observed in HLRCC-associated RCC^11^, would be of substantial importance, thereby confirming that Knudson’s two-hit theory for tumor suppressor genes can be applied to *FH*. Of note, the minor existence of the wild-type allele in the tumor (Fig. 2e) was thought to be attributable to tumor heterogeneity or contamination of normal cells, including capillary endothelial cells within the dissected tumor sample.

In summary, we report a novel pathogenic variant of *FH* in a family with HLRCC. Identification of an underlying genetic cause in the patient helped us to provide family members with precise genetic counseling based on proper assessment of the genetic risk and appropriate surveillance programs in the future. Considering the documented childhood onset and aggressive phenotype of HLRCC-associated RCC, genetic testing and early intervention are crucial for at-risk family members of HLRCC.

## HGV Database

The relevant data from this Data Report are hosted at the Human Genome Variation Database at 10.6084/m9.figshare.hgv.3116.

## Supplementary information

Supplementary Information
