# Supplementary material for: A novel pathogenic variant of the FH gene in a family with hereditary leiomyomatosis and renal cell carcinoma
Source: Hum Genome Var. 2022 Jan 17;9:3. doi: 10.1038/s41439-021-00180-8 (PMC8761746; doi:10.1038/s41439-021-00180-8)
Supplement: Supplementary file 1 — Supplementary Information [file 41439_2021_180_MOESM1_ESM.pdf]

Supplementary Methods

DNA was extracted from peripheral blood leukocytes and macro-dissected renal tumor tissues fixed in formalin and embedded in paraffin using QIAamp DNA Mini kit (Qiagen). Germline multigene panel sequencing was conducted as previously described[1], with a custom-designed panel named NCC Oncopanel FC v2.0 (Agilent) targeting 147 cancer-susceptibility genes including *FH*. Multiplex Ligation-dependent Probe Amplification (MLPA) analysis using SALSA MLPA Probemix P198-A4 FH (MRC-Holland) was added for the detection of copy number changes in the *FH* gene. For Sanger sequencing analysis, exons 1–10 of the *FH* gene were PCR-amplified using previously described primer pairs[2], and then sequenced using the Applied Biosystems 3730xl DNA analyzer (Thermo Fisher Scientific). Written informed consent was obtained from the patient and family members. This research was approved by the local institutional review board.

References

1. Yoshihama, T., Hirasawa, A., Sugano, K., Yoshida, T., Ushiama, M., Ueki, A. *et al.* Germline multigene panel testing revealed a BRCA2 pathogenic variant in a patient with suspected Lynch syndrome. *Int. Cancer Conf. J.* **10**, 6-10 (2021).
2. Furuya, M., Iribe, Y., Nagashima, Y., Kambe, N., Ohe, C., Kinoshita, H. *et al.* Clinicopathological and molecular features of hereditary leiomyomatosis and renal cell cancer-associated renal cell carcinomas. *J. Clin. Pathol.* **73**, 819-825 (2020).

Supplementary Table S1

Clinical and genetic features of Japanese patients with hereditary leiomyomatosis and renal cell carcinoma reported to date.

Family no.	Age (y) at diagnosis	Gender	Manifestations	*FH* variant	Amino acid substitution	Reference
1	70	F	CLM, ULM	c.998G>A	p.Cys333Tyr	1
	51	F	CLM, ULM	NA		
2	39	F	RCC	c.703C>T	p.His235Tyr	2, 3
	58	F	RCC, ULM	NA		
	48	F	RCC, ULM	c.703C>T	p.His235Tyr	
	42	M	RCC	c.703C>T	p.His235Tyr	
3	47	F	CLM, ULM	c.781A>T	p.Arg261Ter	4
4	64	M	RCC	c.1021G>A	p.Asp341Asn	5
	39	M	RCC	c.1021G>A	p.Asp341Asn	
5	20	M	CLM	c.738+2T>A		6
6	32	M	RCC	c.251_267+7del		7
7	34	F	RCC, ULM	c.675del	p.Phe225Leufs*31	8
	28	F	RCC, ULM	NA		
8	34	M	CLM	c.698G>A	p.Arg233His	9
9	34	M	RCC, CLM	c.379-2A>G		10
	62	M	CLM	c.379-2A>G		
	68	F	ULM	c.379-2A>G		
10	42	F	RCC, ULM	c.584T>C	p.Met195Thr	11
11	49	M	RCC	c.641_642del	p.Leu214Serfs*2	12
12	34	M	RCC, CLM	c.1229C>T	p.Pro410Leu	13
13	34	F	RCC, ULM	c.566A>G	p.Asp189Gly	13
14	49	M	RCC	c.641_642del	p.Leu214Serfs*2	13
15	59	M	RCC	c.1067T>C	p.Leu356Ser	13
16	50	M	RCC	c.77C>T	p.Pro26Leu	13
17	44	M	RCC	c.1002T>G	p.Ser334Arg	13
18	58	M	RCC, CLM	c.431G>C	p.Gly144Ala	13
19	7	M	RCC	c.378+1G>A		14
20	35	F	RCC	whole deletion		15

F, female; M, male; CLM, cutaneous leiomyoma; ULM, uterine leiomyoma; RCC, renal cell carcinoma; NA, not available.

References

1. Makino, T., Nagasaki, A., Furuichi, M., Matsui, K., Watanabe, H., Sawamura, D. *et al.* Novel mutation in a fumalate hydratase gene of a Japanese patient with multiple cutaneous and uterine leiomyomatosis. *J. Dermatol. Sci.* **48**, 151-3 (2007).
2. Kamai, T., Tomosugi, N., Abe, H., Kaji, Y., Oyama, T. & Yoshida, K. Protein profiling of blood samples from patients with hereditary leiomyomatosis and renal cell cancer by surface-enhanced laser desorption/ionization time-of-flight mass spectrometry. *Int. J. Mol. Sci.* **13**, 14518-32 (2012).
3. Kamai, T., Abe, H., Arai, K., Murakami, S., Sakamoto, S., Kaji, Y. *et al.* Radical nephrectomy and regional lymph node dissection for locally advanced type 2 papillary renal cell carcinoma in an at-risk individual from a family with hereditary leiomyomatosis and renal cell cancer: a case report. *BMC Cancer* **16**, 232 (2016).
4. Akita, F., Kambe, N., Nakano, M., Satoh, T., Iwasawa, M., Togawa, Y. *et al.* Novel R218X mutation in the fumarate hydratase gene in a patient with Reed's syndrome. *J. Dermatol.* **40**, 58-9 (2013).
5. Kuwada, M., Chihara, Y., Lou, Y., Torimoto, K., Kagebayashi, Y., Tamura, K. *et al.* Novel missense mutation in the FH gene in familial renal cell cancer patients lacking cutaneous leiomyomas. *BMC Res. Notes* **7**, 203 (2014).
6. Yoshinaga, Y., Nakai, H., Hayashi, R., Ito, A., Kariya, N., Ito, M. *et al.* Novel splice site mutation in the fumarate hydratase (FH) gene is associated with multiple cutaneous leiomyomas in a Japanese patient. *J. Dermatol.* **43**, 85-91 (2016).
7. Matsumoto, K., Udaka, N., Hasumi, H., Nakaigawa, N., Nagashima, Y., Tanaka, R. *et al.* Histopathological analysis of aggressive renal cell carcinoma harboring a unique germline mutation in fumarate hydratase. *Pathol. Int.* **68**, 473-478 (2018).
8. Noguchi, G., Furuya, M., Okubo, Y., Nagashima, Y., Kato, I., Matsumoto, K. *et al.* Hereditary leiomyomatosis and renal cell cancer without cutaneous manifestations in two Japanese siblings. *Int. J. Urol.* **25**, 832-835 (2018).
9. Otake, E., Hayama, K., Fujita, H., Shinkuma, S., Shimizu, H. & Terui, T. Leiomyomatosis developed in a patient of neurofibromatosis type 1 with fumarate hydratase gene mutation. *J. Dermatol.* **46**, e456-

e457 (2019).

10. Matsuda, T., Kambe, N., Ly, N.T.M., Ueda-Hayakawa, I., Yamazaki, F., Ohe, C. *et al.* Hereditary leiomyomatosis and renal cell cancer syndrome in which skin biopsy enabled diagnosis. *J. Dermatol.* **46**, e285-e287 (2019).
11. Yonamine, T., Kaname, T., Chinen, Y., Tamashiro, K., Kosuge, N. & Saito, S. Hereditary leiomyomatosis and renal cell cancer (HLRCC): A case report. *Urol. Case Rep.* **30**, 101141 (2020).
12. Iribe, Y., Furuya, M., Shibata, Y., Yasui, M., Funahashi, M., Ota, J. *et al.* Complete response of hereditary leiomyomatosis and renal cell cancer (HLRCC)-associated renal cell carcinoma to nivolumab and ipilimumab combination immunotherapy by: a case report. *Fam. Cancer* **20**, 75-80 (2021).
13. Furuya, M., Iribe, Y., Nagashima, Y., Kambe, N., Ohe, C., Kinoshita, H. *et al.* Clinicopathological and molecular features of hereditary leiomyomatosis and renal cell cancer-associated renal cell carcinomas. *J. Clin. Pathol.* **73**, 819-825 (2020).
14. Taniguchi, R., Muramatsu, H., Okuno, Y., Yoshida, T., Wakamatsu, M., Hamada, M. *et al.* A patient with very early onset FH-deficient renal cell carcinoma diagnosed at age seven. *Fam. Cancer*; e-pub ahead of print 22 June 2021; doi: 10.1007/s10689-021-00268-8.
15. Ueki, A., Sugano, K., Misu, K., Aimono, E., Nakamura, K., Tanishima, S. *et al.* Germline Whole-Gene Deletion of FH Diagnosed from Tumor Profiling. *Int. J. Mol. Sci.* **22**, 7962 (2021).
